# Overexpressed VDAC1 in breast cancer as a novel prognostic biomarker and correlates with immune infiltrates

**DOI:** 10.1186/s12957-022-02667-2

**Published:** 2022-06-22

**Authors:** Yutong Fang, Junpeng Liu, Qunchen Zhang, Chuanghong She, Rongji Zheng, Rendong Zhang, Zexiao Chen, Chunfa Chen, Jundong Wu

**Affiliations:** 1 The Breast Center, Cancer Hospital of Shantou University Medical College, 7 Raoping Rd, Shantou, 515041 Guangdong China; 2 The Department of Central Laboratory, Cancer Hospital of Shantou University Medical College, Shantou, 515041 Guangdong China; 3 Shantou University Medical College, Shantou, 515041 Guangdong China

**Keywords:** Breast cancer, VDAC1, Diagnosis, Prognosis, Immune cells, Bioinformatics

## Abstract

**Background:**

More and more evidence suggests that cancer is a mitochondrial metabolic disease recently and mitochondria dysfunction is critical to tumorigenesis. As a gatekeeper of mitochondria, the voltage-dependent anion channel 1 (VDAC1) is associated with the development of breast cancer (BC). However, its potential mechanism and clinical significance remain unclear; thus, in this research, we aimed to explore it.

**Methods:**

VDAC1 expression in BC tissues and normal tissues was obtained from The Cancer Genome Atlas (TCGA) and validated by datasets from the gene expression omnibus (GEO) database. Then, the relationships between VDAC1 expression and clinicopathological features were analyzed. Receiver operating characteristics (ROC) curves were used to identify the diagnostic value of VDAC1. The prognostic value was evaluated by Kaplan-Meier survival curves and Cox regression analysis. VDAC1 with its co-expression genes were subjected to enrichment analysis to explore potential mechanisms in BC and the protein-protein interaction (PPI) network was constructed. At last, the association between VDAC1 expression and infiltration levels of immune cell infiltration by various methods, as well as their corresponding markers, was analyzed. We also analyzed the correction between VDAC1 expression and eight immune checkpoint genes and the tumor immune dysfunction and exclusion (TIDE) scores of each BC sample in TCGA were calculated and the differences between high and low VDAC1 expression groups were analyzed.

**Results:**

VDAC1 expression was remarkably elevated in BC (*p* < 0.001), and high expression of VDAC1 was associated with the positive expression of ER (*p* = 0.004), PR (*p* = 0.033), and HER2 (*p* = 0.001). ROC analysis suggested that VDAC1 had diagnosed value in BC. The Kaplan-Meier analysis suggested that higher expression of VDAC1 was associated with shorter overall survival (OS), and further Cox regression analysis revealed that VDAC1 was an independent factor of unfavorable prognosis in BC patients. Enrichment analysis of VDAC1 and its co-expression suggested that VDAC1 was related to the regulation of mitochondrial energy metabolism and protein modification, and the HIF-1 singing pathway might be the potential mechanism in BC. Notably, we found that VDAC1 expression was infiltration levels of most types of immune cells, as well as the expression of marker genes of immune cells. The ICGs PDCD1, CTLA4, LAG3, SIGLEC15, and TIGIT were negatively corrected with VDAC1 expression in BC. TIDE scores between the low and high expression groups showed no difference.

**Conclusion:**

Overexpressed VDAC1 in BC could be severed as a novel biomarker for diagnosis and VDAC1 was an independent factor for adverse prognosis prediction. Our study revealed that VDAC1 might inhibit tumor immunity and might be a novel therapeutic target in BC.

## Introduction

Breast cancer (BC) is a public health problem that is plaguing women around the world over the past few decades. In 2020, the global incidence of BC has surpassed lung cancer as the most common malignant tumor in women with approximately 2.26 million new cases [1]. With the advancement of diagnostic and therapeutic strategies, the 5-year survival rate in female patients diagnosed with BC has improved, but the mortality rate of BC is still highest in female cancers [2, 3]. Therefore, early diagnosis and prognosis prediction are crucial for physicians to monitor the disease progression and develop an individualized treatment plan. Currently, early detection of BC mainly relies on some serum biomarkers such as carcinoembryonic antigen (CEA) and carbohydrate antigen 153 (CA153), but their sensitivity and specificity are still low [4, 5]. In addition, there are no effective biomarkers for prognosis prediction of BC currently. Therefore, it is an urgent need for us to identify some novel biomarkers with high sensitivity and specificity for early diagnosis and effective biomarkers for prognosis prediction that is important for optimal treatment planning.

It is well known that mitochondria exist in almost all eukaryotic cells as energy-converting organelles that provide energy to cells through oxidative phosphorylation. Mitochondria are essential to the regulation of cellular energy metabolism, biosynthesis, and cell death, and the dysfunction of which is closely related to the incidence and development of many diseases, including cancers [6]. The voltage-dependent anion channel (VDAC) protein is the most abundant pore-forming protein located in the outer mitochondrial membrane (OMM) of eukaryotic cells working as a gatekeeper of mitochondria that regulate the entry and exit of metabolites, Ca^2+^, fatty acid ions, and reactive oxygen species across the OMM, as well as a hub protein that interacts with other proteins from cytosol and endoplasmic reticulum to regulate cellular metabolism and apoptosis [7–9]. The VDAC protein has been identified as three isoforms encoded by three homologous genes: VDAC1, VDAC2, and VDAC3. VDAC1 has the most abundant expression level among them, VDAC2 is known as an anti-apoptotic protein, and VDAC3 is involved in the ciliary disassembly [10]. It has been reported that the overexpression of VDAC1 is related to many diseases including neurodegeneration, cardiovascular diseases, type 2 diabetes, and different types of cancers. VDAC1 might be served as a novel pharmacological target for anti-cancer therapeutics [8, 9, 11].

However, the evidence about the correlation between the expression level of VDAC1 and BC has been rarely reported and remains unclear, which deserves further exploration. Therefore, the purpose of this study was to analyze the relationships between VDAC1 expression and clinicopathological features, diagnosis value, and prognosis value in BC patients utilizing various online databases. In addition, the co-expression genes of VDAC1 and the relationships between VDAC1 expression and tumor-infiltrating immune cells, as well as their corresponding gene markers, would be analyzed, which would explore the potential mechanism of VDAC1’s role involved in the incidence and development of BC.

## Materials and methods

### Data collection and gene expression analysis

The expression levels of VDAC1 in different types of cancers were analyzed by the Tumor Immune Estimation Resource (TIMER) database (https://cistrome.shinyapps.io/timer/) [12] with the data from The Cancer Genome Atlas (TCGA). The data of the VDAC1 expression in BC patients were downloaded from TCGA (https://portal.gdc.cancer.gov/), including 1065 tumor samples and 111 normal samples, and then the data was converted to as log2 Transcripts Per Million (TPM). Three datasets (GSE21422 [13], GSE33447 [14], and GSE31192 [15]) were selected as validation sets from the Gene Expression Omnibus (GEO) database (https://www.ncbi.nlm.nih.gov/). The expression levels of VDAC1 between normal and tumor groups were analyzed by *T*-test. All of the *p* values in our study less than 0.05 was considered statistically significant. In addition, the figures regarding the immunohistochemistry of VDAC1 expression in human BC tissues and normal tissues were obtained from the Human Protein Atlas (https://www.proteinatlas.org/).

### Analysis of the clinicopathological features and diagnostic value

Data of the clinicopathological features and expression levels of VDAC1 in BC patients were downloaded from TCGA and divided into the low-expression group and high-expression group according to the median expression value of VDAC1. The clinicopathological features are depicted in Table 1. ***T***-test or Kruskal-Wallis test was performed to analyze the different expression levels of VDAC1 among different groups. Moreover, the receiver operating characteristics (ROC) curves were then performed to identify the diagnosis value of VDAC1 to distinguish the normal group and the tumor group, as well as the groups with different types of BC.

**Table 1:** Clinicopathological features of BC patients from TCGA dataset

Clinicopathological features	Low expression of VDAC1	High expression of VDAC1
*n*	532	533
Age, *n* (%)		
≤60	297 (27.9%)	291 (27.3%)
>60	235 (22.1%)	242 (22.7%)
T stage, *n* (%)		
T1	147 (13.8%)	128 (12.1%)
T2	294 (27.7%)	321 (30.2%)
T3	68 (6.4%)	69 (6.5%)
T4	23 (2.2%)	12 (1.1%)
N stage, *n* (%)		
N0	258 (24.7%)	249 (23.8%)
N1	180 (17.2%)	169 (16.2%)
N2	52 (5%)	64 (6.1%)
N3	34 (3.3%)	40 (3.8%)
M stage, *n* (%)		
M0	431 (47.4%)	458 (50.4%)
M1	9 (1%)	11 (1.2%)
Pathologic stage, *n* (%)		
Stage I	94 (9%)	86 (8.3%)
Stage II	308 (29.6%)	298 (28.6%)
Stage III	111 (10.7%)	127 (12.2%)
Stage IV	9 (0.9%)	9 (0.9%)
ER status, *n* (%)		
Negative	138 (13.6%)	99 (9.7%)
Positive	375 (36.9%)	403 (39.6%)
PR status, *n* (%)		
Negative	187 (18.4%)	151 (14.9%)
Positive	323 (31.8%)	351 (34.5%)
HER2 status, *n* (%)		
Negative	293 (40.9%)	255 (35.6%)
Positive	62 (8.6%)	95 (13.2%)

### Overall survival analysis

As noted above, the samples were divided into two groups according to the expression level of VDAC1. The overall survival (OS) curves were visualized by Kaplan-Meier analysis and log-rank test. Then, we further downloaded two datasets (GSE1456 [16] and GSE159956) from the GEO database to validate the OS curves. In addition, univariate and multivariate Cox regression analyses were used to calculate death hazard ratios of clinicopathological features and VDAC1 expression and identify whether VDAC1 could be served as an independent prognostic factor for BC.

### Identification and enrichment analysis of co-expression genes

The co-expression genes of VDAC1 in the data of BC from TCGA were identified via the database LinkedOmics (http://www.linkedomics.org/login.php) [17] and Pearson correlation test. Then, we selected the top 200 co-expression genes to perform Gene Ontology (GO) and Kyoto Encyclopedia of Genes and Genomes (KEGG) pathway enrichment analysis by the online database Metascape (http://metascape.org) [18] to explore the VDAC1-related molecular mechanisms. We set the minimum counts larger than 3, *p*-value less than 0.05, and minimum enrichment factors larger than 1.5 as thresholds. Moreover, the top 200 co-expression genes were uploaded to the STRING database (https://cn.string-db.org/) [19] for the construction of the protein-protein interaction (PPI) network with a minimum required interaction score of 0.9 and then visualized by the gene-networking tool Cytoscape (version 3.8.2).

### Immune cell analysis

The single sample GSEA method from the R package “GSVA” [20] was applied to present infiltration enrichment of 24 common immune cells and the relation between VDAC1 expression with immune cell infiltration was analyzed by the Spearman test. The Microenvironment Cell Populations-counter (MCP-counter) algorithm [21] and the TIMER algorithm were also employed to analyze the correction between VDAC1 expression and immune cell infiltration. The Estimation of Stromal and Immune cells in Malignant Tumor tissues using Expression (ESTIMATE) algorithm [22] was used to calculate and compare stromal scores, immune scores, and ESTIMATE scores between low and high VDAC1 expression groups of BC samples from TCGA dataset by Wilcoxon rank-sum test. Moreover, we further analyzed the association of the expression of VDAC1 in BC with multiple marker genes of immune cells by the TIMER database.

### Immune checkpoint gene analysis and immunity therapy response prediction

Patients with high expression of immune checkpoint inhibitors (ICIs) will receive greater benefits from ICI therapy [23]. We analyzed the correction between VDAC1 expression and eight immune checkpoint genes (ICGs) by the Spearman test, which was SIGLEC15, TIGIT, CD274, HAVCR2, PDCD1, CTLA4, LAG3, and PDCD1LG2. The tumor immune dysfunction and exclusion (TIDE) scores [24] of each BC sample in TCGA were calculated and the differences between high and low VDAC1 expression groups were analyzed by the Wilcoxon rank-sum test to predict ICI therapy response.

## Result

### VDAC1 exhibited higher expression levels in BC than that in normal tissues

**Fig. 1:**
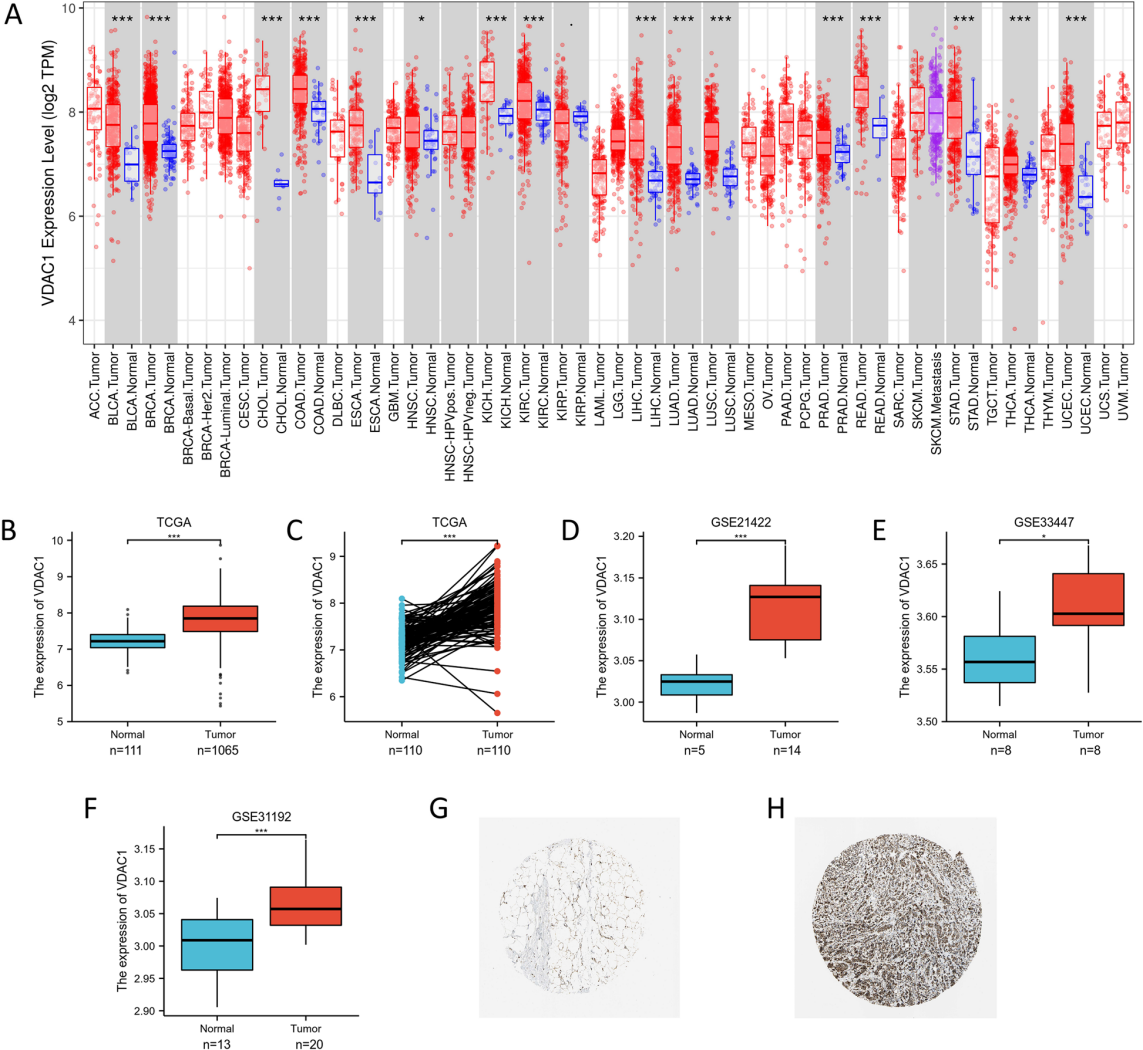
VDAC1 mRNA expression levels in cancers. **A** The comparison of VDAC1 expression in different types of cancers and normal tissues from the TIMER database. **B** VDAC1 expression was significantly increased in BC tissues compared to normal tissues from the TCGA dataset. **C** VDAC1 expression was significantly increased in BC tissues compared to matched normal tissues from the TCGA dataset. **D**–**F** VDAC1 expression was significantly increased in BC tissues compared to normal tissues from the GEO datasets. **G** The expression of VDAC1 was lower in normal breast tissue than **H** breast cancer tissue in the Human Protein Atlas (Antibody CAB005885, 10X). NS indicates no statistical difference, *p < 0.05, **p < 0.01, ***p < 0.001

Initially, we evaluated the expression levels of VDAC1 in different types of human malignant tumors from the TCGA RNA-seq data via the TIMER (Fig. 1A). The expression level of VDAC1 in the BC tissue group was remarkably higher than that in the normal tissue group. Moreover, the expression of VDAC1 was especially higher in human epidermal growth factor receptor 2 (HER2)-enriched BC than that in luminal and basal-like BC tissues. In addition, the result revealed that VDAC1 exhibited significantly higher expression levels in many types of malignant tumors such as bladder cancer (BLCA), cholangiocarcinoma (CHOL), chromophobe renal cell carcinoma (KICH), liver hepatocellular carcinoma (LIHC), lung adenocarcinoma (LUAD), and stomach adenocarcinomas (STAD). Analysis of the expression of VDAC1 expression between BC tissue group and normal tissue group, as well as matched normal tissues, suggested that VDAC1 exhibit remarkably higher expression level in BC (Fig. 1B, C) (*p* < 0.001, respectively). The result was further validated by three datasets from the GEO database (Fig. 1D–F) (*p* < 0.001; *p* = 0.042; *p* = 0.001, respectively). Correspondingly, the immunohistochemistry from the Human Protein Atlas revealed that VDAC1 protein was significantly expressed in BC tissues, especially in cytoplasmic and membranous of BC cells, and rarely expressed in normal tissues (Fig. 1G, H).

### Expression of VDAC1 was associated with ER, PR, and HER2

**Fig. 2:**
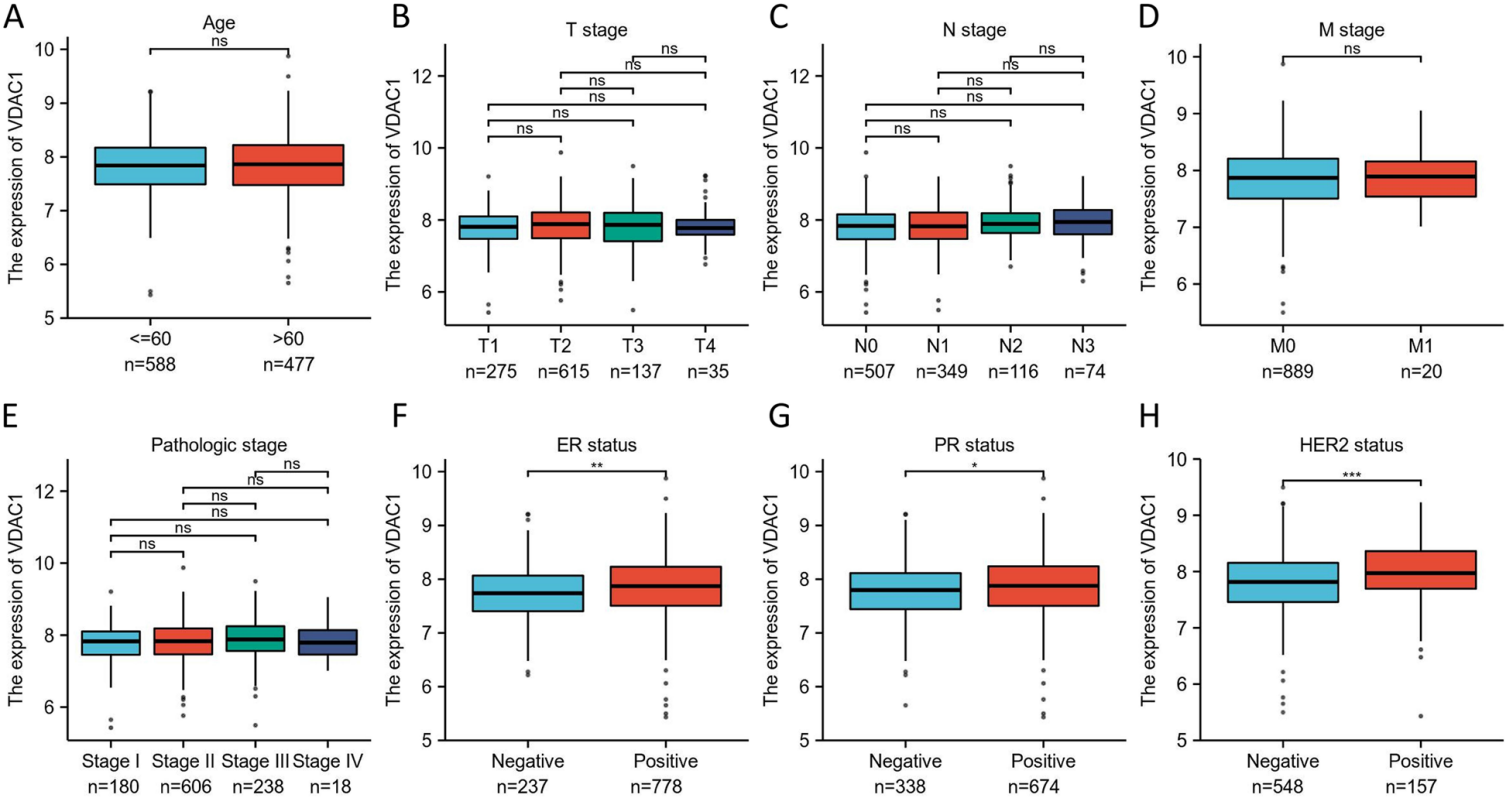
The association of VDAC1 expression with clinicopathological features of BC patients from the TCGA dataset. The clinicopathological features included **A** age, **B** T stage, **C** N stage, **D** M stage, **E** pathologic stages, **F** ER status, **G** PR status, and **H** HER2 status. NS indicates no statistical difference, *p < 0.05, **p < 0.01, ***p < 0.001

The clinicopathological features of the BC patients from the TCGA dataset are shown in Table 1. We analyzed the expression of VDAC1 in BC patients with different clinicopathological features and the samples with indeterminate information were excluded. The result of our analysis shows that there was no significant difference in age (*p* = 0.681), T stages (*p* = 0.328), N stages (*p* = 0.374), M stages (*p* = 0.980), and pathologic stages (*p* = 0.282). The high expression of VDAC1 was correlated with the positive expression of estrogen receptor (ER) (*p* = 0.004), progesterone receptor (PR) (*p* = 0.033), and HER2 (*p* = 0.001) (Fig. 2).

### VDAC1 could be served as a biomarker for BC diagnosis

**Fig. 3:**
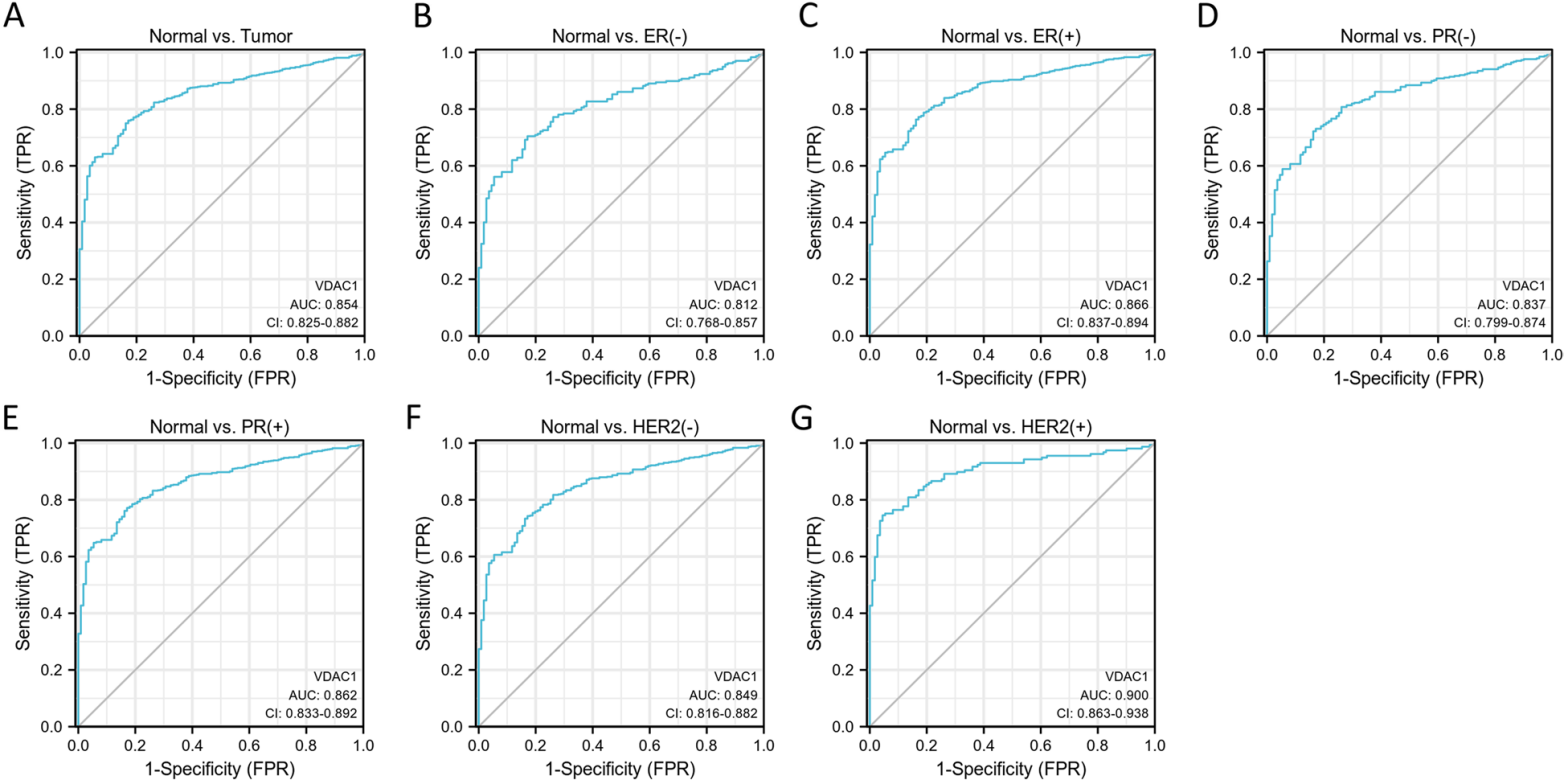
ROC curve analysis of VDAC expression in **A** BC, **B** ER (-), **C** ER (+), **D** PR (-), **E** PR (+), **F** HER2 (-), and **G** HER2 (+) BC group to discriminate from the normal group

The ROC curves were used to identify the effectiveness of VDAC1 mRNA expression level to distinguish BC tissues from the normal tissues. The area under the curve (AUC) was 0.854 [95% confidence interval (CI): 0.825–0.882], with 76.0% sensitivity and 82.9% specificity (Fig. 3A). The expression of VDAC1 was validated to be associated with the status of ER, PR, and HER2 in the above findings; thus, we further evaluate the diagnostic value of VDAC1 in negative and positive expression groups of ER, PR, and HER2, respectively. In the comparison of ER-negative expression and normal tissue groups, the AUC was 0.812 (95% CI: 0.768–0.857), with 70.5% sensitivity and 82.9% specificity (Fig. 3B). In the comparison of ER-positive expression and normal tissue groups, the AUC was 0.866 (95% CI: 0.837–0.894), with 76.3% sensitivity and 83.8% specificity (Fig. 3C). The AUC of VDAC1 was 0.837 (95% CI: 0.799–0.874 ) with 72.2% sensitivity and 83.8% specificity (Fig. 3D) in the comparison between PR-negative expression and normal tissue groups and the AUC was 0.863 in the comparison between PR-positive and normal tissue groups (95% CI: 0.833–0.892) with 77.2% sensitivity and 82.9% specificity (Fig. 3E). The AUC of VDAC1 was 0.849 (95% CI: 0.816–0.882) with 74.3% sensitivity and 82.9% specificity in the comparison between HER-negative expression and normal tissue groups (Fig. 3F). Intriguingly, we found that VDAC1 exhibits the best effectiveness to distinguish HER2-positive tumor tissues from the normal tissues, the AUC of which was 0.900 (95% CI: 0.863–0.938) with 74.5% sensitivity and 95.5% specificity (Fig. 3G).

### VDAC1 was an independent factor for prognosis prediction in BC

**Fig. 4:**
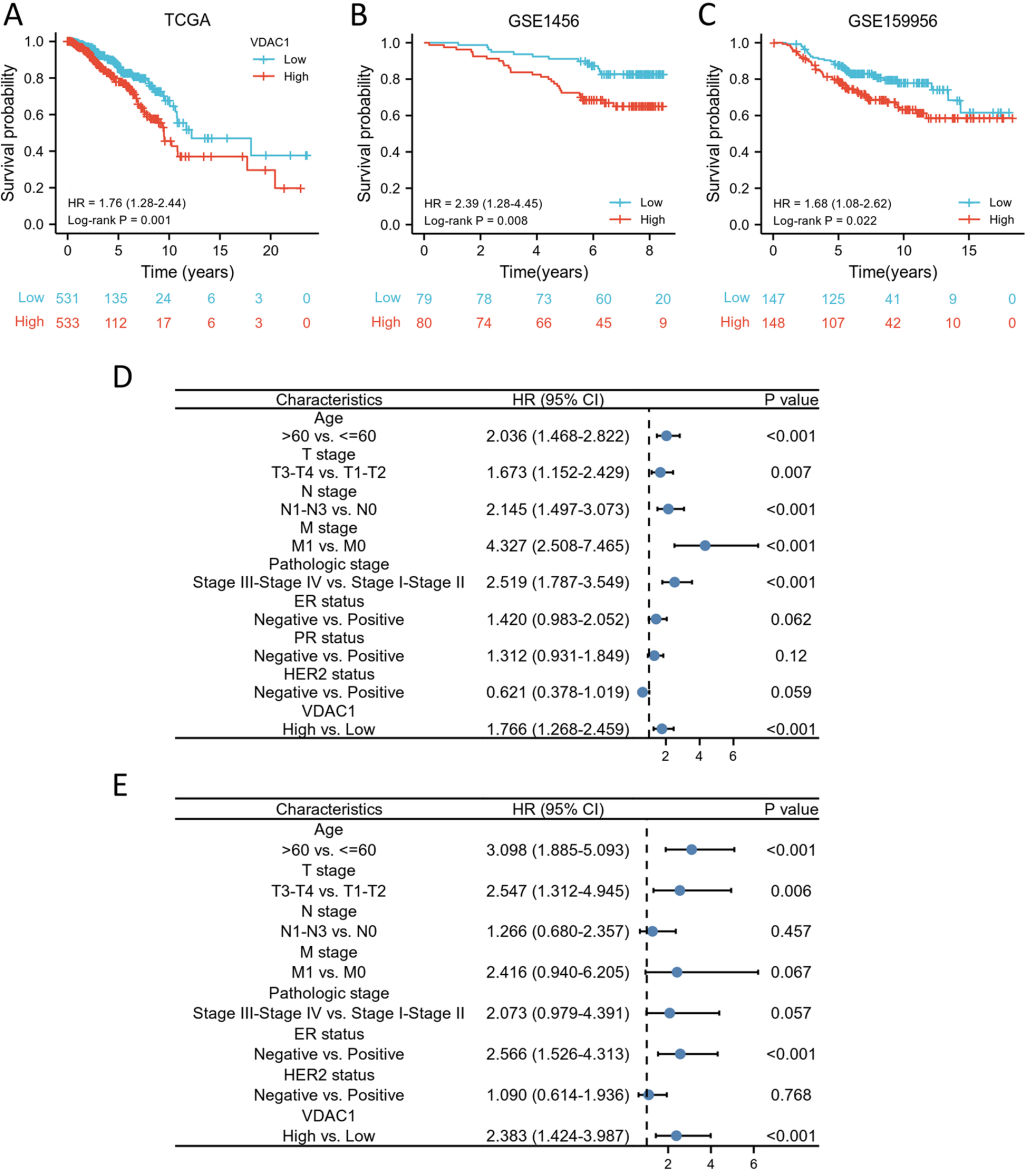
High VDAC expression is associated with poor OS in BC patients. **A** The Kaplan-Meier survival curves of the BC patients with high and low VDAC1 expression level form TCGA and **B**, **C** validation datasets from the GEO database. **D** Univariate and **E** multivariate regression analysis of VDAC1 and clinicopathological features with OS in BC patients from TCGA

To identify the correlation between VDAC1 expression and the OS of BC patients from TCGA, the survival curves were visualized by Kaplan-Meier analysis and log-rank test. As indicated in Fig. 4A, higher VDAC1 mRNA expression was remarkably related to shorter OS (HR = 1.76, *p* = 0.001). The result was validated by two datasets from GEO (Fig. 4B, C).

Cox regression analysis was further performed to evaluate the prognostic value of VDAC1 in BC. The *p* values of the clinicopathological features less than 0.1 in the univariate Cox model were considered statistically significant and the features were then further put into multivariate Cox analysis. As indicated in Fig. 4D, OS of BC patients was significantly correlated with age, T stages, N stages, pathologic stages, ER status, HER2 status, and VDAC1 expression. In the multivariate Cox model, the clinicopathological features including age, T stage, and ER expression, as well as VDAC1 expression, were independent predictors for poor prognosis prediction in BC (Fig. 4E). Overall, our results revealed that VDAC1 was an independent factor of unfavorable prognosis in BC patients.

### Enrichment analysis and network establishment for co-expression genes of VDAC1 in BC

**Fig. 5:**
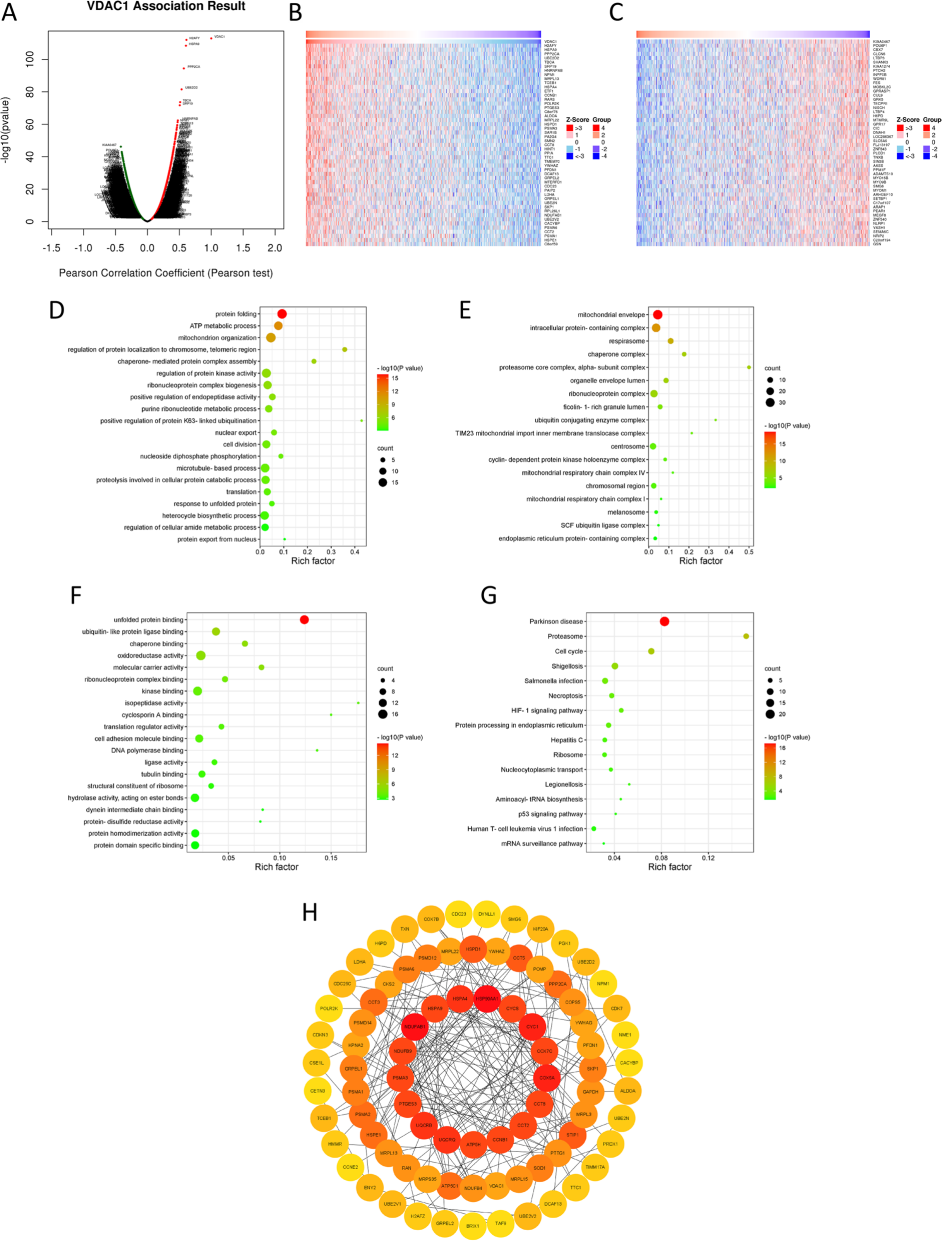
Analysis of VDAC1 and its co-expression genes in BC. **A** Highly corrected genes with VDAC1 identified by the Pearson test. Red and green dots represent genes positively and negatively corrected with VDAC1, respectively. **B** Top 50 positively and **C** negatively corrected genes with VDAC1. GO analysis in **D** biological process, **E** cellular component and **F** molecular functions ontology and KEGG pathway enrichment analysis of VDAC1 and its corrected genes (**G**). **H** PPI network of VDAC1 and its corrected genes

To explore the possible mechanism and biological function of VDAC1’s role involved in BC, we identified the co-expression genes of VDAC1 in BC via the online database LinkedOmics. As shown in Fig. 5A, a total of 10,651 genes were significantly corrected with the expression of VDAC1, including 3651 positively corrected genes (red dots) and 7000 negatively corrected genes (green dots), and the false discovery rate (FDR) was less than 0.01. The top 50 positively and negatively corrected genes were shown in two heatmaps (Fig. 5B, C), respectively.

The top 200 co-expression genes and VDAC1 were then selected for further GO and KEGG pathway enrichment analysis by the Matascape. The results of GO analysis suggested that VDAC1 and its related genes are remarkably enriched in protein folding, ATP metabolic process, mitochondrion organization, etc. in the biological process (BP) ontology; mitochondrial envelope, intracellular protein-containing complex, respirasome, etc. in the cellular component (CC) ontology; and unfolded protein binding, etc. in the molecular function (MF) ontology (Fig. 5D–G**)**. KEGG pathway enrichment analysis showed that Parkinson disease, proteasome, and cell cycle were the most enriched pathway.

In addition, the top 200 co-expression genes and VDAC1 were uploaded to STRING for construction of the PPI network and then were visualized via the gene-networking tool Cytoscape (Fig. 5H). The PPI network contained 84 nodes and 207 edges, in which VDAC1 was the hub gene that related to another 4 genes.

### VDAC1 was correlated with tumor-infiltrating immune cells in BC

**Fig. 6:**
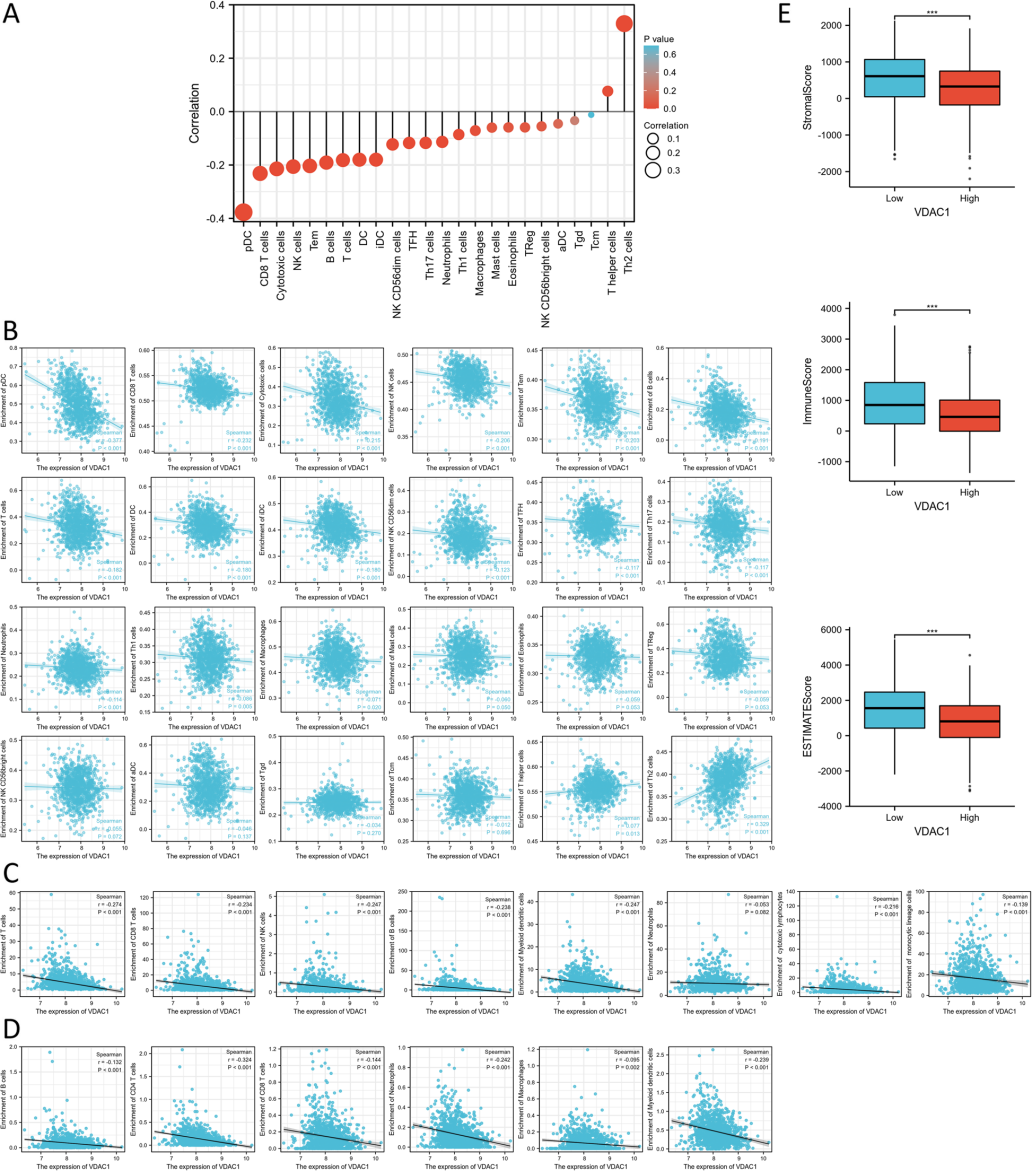
Correlation of VDAC1 expression and infiltration levels of immune cells in BC patients. **A** The relation between VDAC1 expression with 24 common immune cells infiltration analyzed by ssGSEA. **B** Scatter plots depicting the association between VDAC1 expression and 24 immune cell infiltration analyzed by ssGSEA. **C** Scatter plots depicting the association between VDAC1 expression and infiltration of immune cells analyzed by MCP-counter. **D** Scatter plots depicting the association between VDAC1 expression and infiltration of immune cells analyzed by TIMER. **E** Box diagram showing the stromal scores, immune scores, and ESTIMATE score in the low and high VDAC1 expression groups analyzed by the ESTIMATE algorithm

Firstly, we used the ssGSEA method to present infiltration enrichment of 24 common types of immune cells in BC. Subsequently, the relation between VDAC1 expression with immune cell infiltration was analyzed by Spearman’ analysis. A total of 24 immune cells and their relationships with VDAC1 expression are shown in Fig. 6A and 17 types of immune cells were significantly correlated with VDAC1 expression. There were 15 types of immune cells negatively corrected with VDAC1 expression, which were plasmacytoid DCs (pDCs) (*r* = −0.377, *p* = 2.60e−37), CD8 + T cells (*r* = −0.232, *p* = 2.34e−14), cytotoxic cells (*r* = −0.215, *p* = 1.71e−12), natural killer (NK) cells (*r* = −0.206, *p* = 1.24e−11), T effector memory (Tem) cells (*r* = −0.203, *p* = 2.30e−11), B cells (*r* = −0.191, *p* = 3.53e−10), T cells (*r* = −0.182, *p* = 2.45e−09), dendritic cells (DCs) (*r* = −0.180, *p* = 3.33e−09), immature DCs (iDCs) (*r* = −0.180, *p* = 3.45e−09), NK 56-cells (*r* = −0.123, *p* = 5.84e−05), T follicular helper (Tfh) cells (*r* = −0.117, *p* = 1.23e−04), type 17 Th (Th17) cells (*r* = −0.117, *p* = 1.26e−04), neutrophils (*r* = −0.114, *p* = 2.08e−04), type 1 Th (Th1) cells (*r* = −0.086, *p* = 0.005), and macrophages (*r* = −0.071, *p* = 0.020). Two types of immune cells positively corrected with VDAC1 expression were type 2 Th (Th2) cells (*r* = 0.329, *p* = 0.000), and T helper (Th) cells (*r* = 0.077, *p* = 0.013) (Fig. 6B). The results of the MCP-counter method suggested that VDAC1 expression was negatively corrected with seven kinds of immune cells (Fig. 6C) and the TIMER algorithm suggested that VDAC1 expression was negatively corrected with infiltrating levels of six common immune cells (Fig. 6D). The ESTIMATE algorithm suggested that the high-VDAC1 group had lower immune scores (*p* < 0.001), stromal scores (*p* < 0.001), and ESTIMATE scores (*p* < 0.001) than the low-VDAC1 group (Fig. 6E).

### Correction between the expression of VDAC1 and marker genes of immune cells in BC

We further analyzed the association between the expression of VDAC1 and various immune signatures of various immune cells via TIMER databases. A total of 60 immune marker genes were analyzed, which were well accepted as corresponding markers of different types of immune cells, such as B cells, macrophages, neutrophils, monocytes, NK cells, dendritic cells, and different functional T cells. Our results revealed that most immune markers of immune cells were remarkably associated with the expression of VDAC1 in BC (Table 2).

**Table 2:** Relationships of VDAC1 expression and marker genes of immune cells in TIMER

Cell type	Gene marker	None		Purity	
		Cor	*p*	Cor	*p*
B cell	CD19	−0.154	***	−0.083	**
	CD20(KRT20)	0.114	***	0.126	***
	CD38	−0.073	*	0.011	0.719
CD8 + T cell	CD8A	−0.125	***	−0.04	0.203
	CD8B	−0.191	***	−0.111	***
Tfh	BCL6	−0.067	*	−0.033	0.305
	ICOS	−0.001	0.967	0.092	**
	CXCR5	−0.176	***	−0.097	**
Th1	T-bet (TBX21)	−0.174	***	−0.096	**
	STAT4	−0.104	***	−0.001	0.970
	IL12RB2	−0.033	0.269	0.029	0.357
	WSX1(IL27RA)	−0.245	***	−0.191	***
	STAT1	0.173	***	0.218	***
	IFN-γ (IFNG)	−0.091	**	−0.024	0.449
	TNF-α (TNF)	−0.059	0.050	−0.01	0.762
Th2	GATA3	0.167	***	0.116	***
	CCR3	−0.049	0.103	−0.011	0.720
	STAT6	−0.064	*	−0.055	0.082
	STAT5A	−0.165	***	−0.124	***
Th9	TGFBR2	−0.07	*	0.036	0.261
	IRF4	−0.124	***	−0.024	0.454
	PU.1(SPI1)	−0.226	***	−0.172	***
Th17	STAT3	0.107	***	0.117	***
	IL-21R	−0.108	***	−0.015	0.636
	IL-23R	−0.02	0.505	0.038	0.233
	IL-17A	−0.055	0.067	−0.019	0.559
Th22	CCR10	−0.314	***	−0.293	***
	AHR	0.074	*	0.131	***
Treg	FOXP3	−0.003	0.909	0.087	**
	CD25(IL2RA)	0.007	0.814	0.099	**
	CCR8	0.153	***	0.223	***
T cell exhaustion	PD-1 (PDCD1)	−0.178	***	−0.112	***
	CTLA4	−0.057	0.058	0.026	0.419
	LAG3	−0.112	***	−0.07	*
	TIM-3 (HAVCR2)	0.009	0.759	0.085	**
Macrophage	CD68	0.000	0.997	0.069	*
	CD11b (ITGAM)	−0.076	*	−0.028	0.378
M1	INOS (NOS2)	−0.115	***	−0.107	***
	IRF5	0.006	0.850	0.025	0.430
	COX2(PTGS2)	−0.133	***	−0.058	0.066
M2	CD163	0.023	0.446	0.088	**
	ARG1	−0.025	0.411	0.026	0.405
	MRC1	−0.039	0.198	0.048	0.134
	MS4A4A	0.006	0.844	0.09	**
TAM	CCL2	−0.08	**	−0.009	0.767
	CD80	0.122	***	0.177	***
	CD86	−0.012	0.692	0.068	*
	CCR5	−0.073	*	0.022	0.491
Monocyte	CD14	−0.225	***	−0.181	***
	CD16(FCGR3B)	0.141	***	0.163	***
	CD115 (CSF1R)	−0.186	***	−0.123	***
Neutrophil	CD66b (CEACAM8)	−0.101	***	−0.099	**
	CD15(FUT4)	−0.09	**	0.002	0.952
	CD11b (ITGAM)	−0.076	*	−0.028	0.378
Natural killer cell	XCL1	−0.138	***	−0.059	0.064
	CD7	−0.204	***	−0.141	***
	KIR3DL1	−0.091	**	−0.034	0.280
Dendritic cell	CD1C(BDCA-1)	−0.217	***	−0.149	***
	CD141(THBD)	−0.136	***	−0.101	**
	CD11c (ITGAX)	−0.101	***	−0.033	0.305

### Correction between the expression of VDAC1 and ICGs in BC

**Fig. 7:**
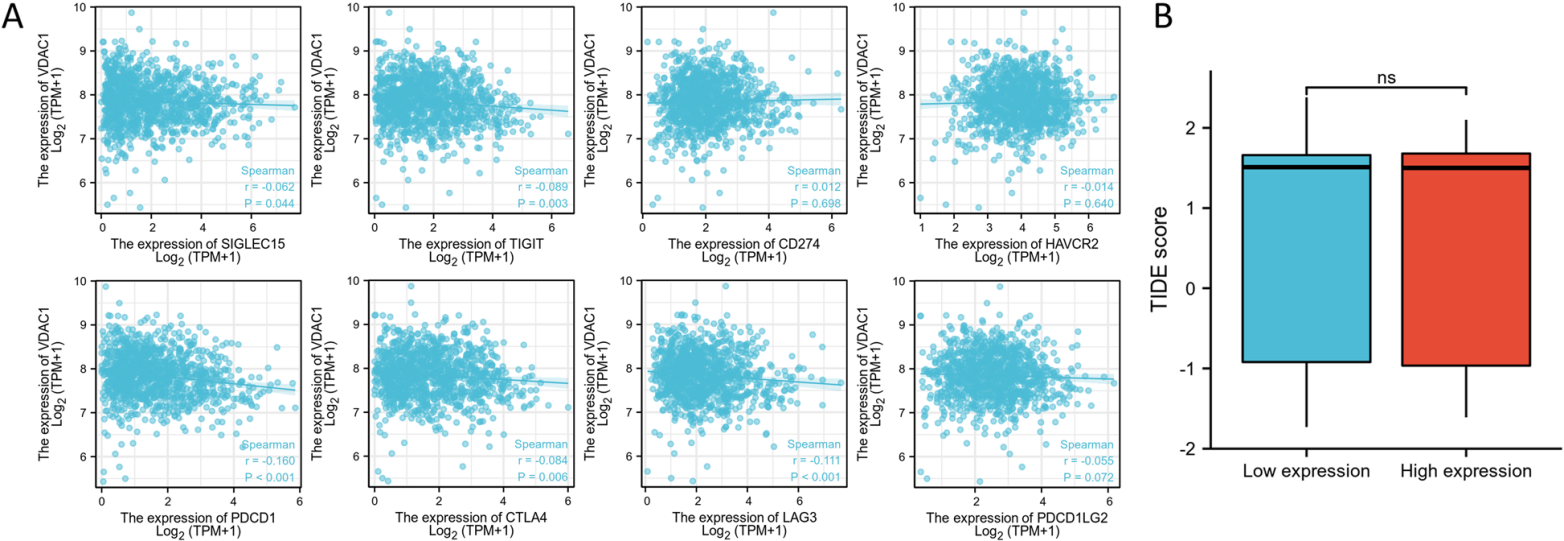
**A** Scatter plots depicting the correction between VDAC1 expression and eight ICGs. **B** TIDE scores of the low and high VDAC1 groups of BC patients from the TCGA database

At last, we analyzed the association between the expression of VDAC1 and eight ICGs. We found that the expression of VDAC1 was negatively corrected with the expression of PDCD1 (*r* = −0.160, *p* < 0.001), CTLA4 (*r* = −0.084, *p* = 0.006), LAG3 (*r* = −0.111, *p* < 0.001), SIGLEC15 (*r* = −0.062, *p* = 0.044), and TIGIT (*r* = −0.089, *p* = 0.003) (Fig. 7A). Further analysis showed that there was no difference in the TIDE score between the high-VDAC1 group and the low-VDAC1 group (*p* = 0.798) (Fig. 7B) despite the proportion of patients who responded to ICI therapy in the high-expression group (37.1%) was higher than in the low-expression group (33.2%).

## Discussion

Breast cancer is the most common malignant tumor in women. The incidence of young patients diagnosed with BC has been rising over the past decade [25]. Many biomarkers have been proven to be involved in the BC progression and associated with the prognosis of BC patients [26–29]. The prognosis role of cancer driver genes and methylated genes in BC have also been reported [30, 31]. Interestingly, a high systemic immune-inflammation index has been reported to predict the poor prognosis of BC patients as a promising indicator [32]. In addition, absolute lymphocyte count and insulin resistance have been reported to be served as predictors for chemotherapy response [33, 34]. Tang et al. have successfully developed a nomogram to predict pathological complete response after neoadjuvant chemotherapy of ER-positive and HER2-negative BC patients [35]. All in all, in the future, more and more tools would be used as predictors in BC treatment.

In the present study, we explored the clinical significance of a mitochondrial porin, VDAC1, in BC via the datasets obtained from TCGA. We also found that increased expression of VDAC1 was related to the positive status of ER, PR, and especially HER2. We observed that the expression level of VDAC1 was remarkably increased in BC tissues compared to normal controls, which was consistent with a recent study that detected the expression of VDAC1 protein in BC tissues and benign breast lesions via immunohistochemistry [36] and validated by 3 datasets obtained from GEO database. In addition, we found that the expression of VDAC1 was higher in many types of tumor tissues than that in corresponding normal tissues, which was consistent with previous studies [37–39] and suggested that VDAC1 might act as an oncogene that is related to tumorigenesis and tumor progression. Currently, early detection is critical to the treatment of cancers. In our study, we performed ROC analysis and found that VDAC1 was an effective biomarker for the diagnosis of BC patients, especially HER2-positive BC patients. Moreover, in our study, we revealed that high expression of VDAC1 was closely related to a shorter OS rate of BC patients. Cox regression analysis indicated that VDAC1 could be severed as an independent risk factor for poor prognosis of BC patients, which is consistent with previous research [36]. VDAC1 has also been identified with poor outcomes in other types of malignant tumors, such as pancreatic cancer, hepatocellular carcinoma, and cervical cancer [40–42]. These results might support that VDAC1 plays an important role in the development of cancers by different potential mechanisms.

We further attempted to explore the potential mechanisms and functions of VDAC1 involved in BC; thus, we performed GO and KEGG pathway enrichment analysis of co-expression of VDAC1. GO analysis suggested that VDAC1 and its co-expression showed significant enrichment in mitochondrial energy metabolism and protein modification. VDAC1 is a multi-functional channel protein and served as a hub that is involved in the control of cell metabolism, oxidative stress, apoptosis, mtDNA release, and more [8]. Overexpression of VDAC1 has been validated to be associated with cancers [8, 9, 37–40], but its role involved in BC remains unclear. In recent years, more and more evidence suggests that cancer is a mitochondrial metabolic disease, and dysfunction of mitochondria is critical to tumorigenesis [43]. As a gatekeeper of mitochondria, VDAC1 can decide the fate of cancer cells by regulating metabolic and energetic functions. The overexpressed VDAC1 in the cancer cells can interact with hexokinase (HK), a rate-limiting enzyme of glycolysis, and then promote mitochondrial ATP to coupling to glucose, which contributes to cancer cell metabolism [8, 9, 11]. VDAC1 has also been found to be involved in anti-apoptosis by interacting with anti-apoptotic proteins overexpressed in cancer such as Bcl-2, Bcl-xL, HK, and mediating the release of Cytc to prevent the cancer cells from apoptosis [8, 9, 11]. A recent study suggested that the high expression of VDAC1 in BC could be inhibited by the bromodomain inhibitor (JQ1) and associated with the resistance to JQ1 [44]. It has been reported that mitochondrial fission factor (MFF) can bind to VDAC1 and the MFF-VDAC1 complex can be severed as an actionable therapeutic target in BC [45]. Moreover, microRNA-7 was validated to decrease the expression of VDAC1 to inhibit hepatocellular carcinoma proliferation and metastasis [42]. In addition, VDAC1 was found to be upregulated by microRNA-320a to promote proliferation and invasion of non-small cell lung cancer [38]. These findings reveal that VDAC1 could be severed as a novel drug target for the treatment of BC. In our pathway enrichment analysis, the hypoxia-inducible factor 1 (HIF-1) signaling pathway was enriched by VDAC1 co-expression genes. HIF-1 signaling is known to play an important role in the tumor microenvironment; thus, HIF-1 signaling is a promising target for the treatment of cancers [46].

In our study, we also explored the underlying relationship between VDAC1 expression and immune cell infiltration. Our study suggested that VDAC1 expression was negatively corrected with the infiltration levels of most types of immune cells, such as DCs and CD8 + T cells, as well as the expression of their corresponding markers. It is well accepted now that CD8 + T cells are central in mediating anti-cancer immunity and activated by DCs to initiate anti-cancer immunity [47]. VDAC1 expression was negatively corrected with neutrophils and macrophages, which play important roles in anti-cancer immunity [48]. Further analysis of the gene markers of immune cells showed that markers of M2 macrophages such as CD163 and MS4A4A had weak correlations with BICC1 expression. M2 macrophage marker NOS2 and TAM marker CD80 showed moderate relationships with VDAC1 expression, which suggested that VDAC1 might involve in the regulation of the polarization of macrophages. NK cells are a specialized type of immune cells that can kill adjacent cells with surface markers related to oncogenic transformation. In the past decades, the fields of NK cell-based cancer immunotherapy have grown exponentially [49]. The negative correction between VDAC1 expression and NK cell infiltration, as well as their markers CD7, indicated that VDAC1 might inhibit the activation of NK cells. These results suggested that overexpressed VDAC1 seemed to dampen tumor immunity thus contributing to the tumorigenesis and development of BC.

With the development of immunotherapy, ICI therapy has exhibited great efficacy in breast cancer treatment but only a small number of patients benefit from it [50]. In our study, we analyzed the relationship between eight ICGs and VDAC1 expression, among which PDCD1, CTLA4, LAG3, SIGLEC15, and TIGIT were negatively corrected with VDAC1 expression in BC. The results suggested that BC patients with low VDAC1 expression might receive greater benefits from ICI therapy with a better prognosis. However, TIDE scores between low and high expression groups showed no difference. VDAC1 was not a reliable biomarker to predict the response to ICI therapy.

## Conclusion

All in all, our study demonstrated the overexpression of VDAC1 in BC, which might be served as a novel biomarker for the diagnosis of BC patients. High expression of VDAC1 was associated with poor prognosis and VDAC1 was an independent factor for poor outcome prediction of BC patients. High expression of VDAC1 was closely associated with low infiltration levels of most types of immune cells. Our study revealed that VDAC1 might inhibit tumor immunity and might be served as a novel therapeutic target in BC.

## Data Availability

The data underlying this study are freely available from the TCGA dataset (https://portal.gdc.cancer.gov/projects/TCGA-KIRC) and the GEO dataset (http://www.ncbi.nlm.nih.gov/geo/). The authors did not have special access privileges.

## Abbreviations

BC: Breast cancer
CEA: Carcinoembryonic antigen
CA153: Carbohydrate antigen 153
VDAC: Voltage-dependent anion channel
OMM: Outer mitochondrial membrane
TIMER: Tumor Immune Estimation Resource
TCGA: The Cancer Genome Atlas
TPM: Transcripts Per Million
GEO: Gene Expression Omnibus
ROC: Receiver operating characteristics
OS: Overall survival
GO: Gene Ontology
KEGG: Kyoto Encyclopedia of Genes and Genomes
ESTIMATE: Estimation of Stromal and Immune cells in Malignant Tumor tissues using Expression
PPI: Protein-protein interaction
ssGSEA: Single sample GSEA
MCP-counter: Microenvironment Cell Populations-counter
ICI: Immune checkpoint inhibitor
ICGs: Immune checkpoint genes
TIDE: Tumor immune dysfunction and exclusion
HER2: Human epidermal growth factor receptor 2
ER: Estrogen receptor
PR: Progesterone receptor
AUC: Area under the curve
CI: Confidence interval
